# ‘Fertility’ and the Carnival 2: Popular Frazerism and the Reconfiguration of Tradition in Europe Today

**DOI:** 10.1080/0015587X.2017.1281967

**Published:** 2017-05-19

**Authors:** Alessandro Testa

## Abstract

This article examines European festive culture through the lens of two ethnographic case studies of carnivals, conducted in Italy and the Czech Republic. The article analyses processes of meaning construction, cultural circulation, and reconfiguration of local traditions that are currently widely at work in rural and marginal European contexts. It explains why I propose to name the cultural complex shaped by those processes ‘popular Frazerism’. The article also argues that these phenomena are representative of a certain post-modern romantic imaginary of magic, antiquity, and primitiveness, and explores the symbolic sources and the social needs from which this imaginary draws its strengths and legitimacy.

This article is the second part of a broader study divided into two parts.

## Carnivals and Fertility

This article is obviously not the place to propose an exhaustive review of the functionalist interpretation of European carnivals.^1^ Nor is it the place for an assessment of the topic of fertility in folkloristic and anthropological studies. The article will instead focus on two emic^2^ declensions of the notion of magically or ritually induced fertility that I have studied in the literature and observed in two European cases, which have been the objects of my recent historical and ethnographic research. Nonetheless, in order to understand the nature, the implications, and the historical development of these phenomena fully, it is necessary to go through a brief genealogy of the notion of fertility as it has been developed and used to interpret European festive events, and especially carnivals.

The first relevant anthropological account of the origins and function of European seasonal festivals is to be found in the work of Sir James Frazer.^3^ His work on such themes was perhaps not the first, but quite surely the widest and the most systematic, and certainly the most influential. According to Frazer’s interpretation, European carnival traditions, documented from the Middle Ages onwards throughout the continent, were to be included in the macro-category of springtime festivities in which propitiatory^4^ magical rituals were performed for the fertility of the fields. In other words, festivities for the ‘awakening’ or cyclic renewal of nature after the winter.^5^ These traditions presumably had pre-Christian origins and had ‘survived’, according to historical circumstances and especially in rural folklore, in different forms and intensity. As ‘survivals’ of pagan times, they were linked to a deep past, but at the same time still bore a function for the communities that had kept and transmitted them, in different forms, for hundreds of years up until the Victorian century of progress and industrialization.

Frazer was interested not only in carnival features as observable in folklore, but also in their general origins and functions. In particular, his attention focused on the ‘magical’ property of the acts and rituals performed during carnival. For example, he regarded the ritual killing of the carnival puppet, a pseudo-rite widespread throughout Europe, as a particular kind of his famous sympathetic magic. The puppet, often called by grandfather-like nicknames, represented the out-going year (or the winter) that had to be ‘killed’ to eliminate the old and in so doing ensure the coming of the new, the rebirth of nature, and therefore the renewed fertility of the fields.

Another hypothesis found in Frazer’s pages about carnivals and similar festivities, and worth citing here, is also linked to their temporal and calendric dimensions: it is based on the idea that, like other pagan festivals, during European carnivals it was possible to observe a tripartite dynamic of social order. During carnival, the local society went from a pre-festive period of stability through a ritualized disorder and subversion of the normal societal and political order, only to undertake, afterwards, a restoration of that same order.

One can therefore consider Frazer’s interpretations of carnival to be founded on at least three major features: the diachronic dimension or ‘survival’ of these festivities from ancient times; the propitiatory function through sympathetic magic embodied especially—but not exclusively—in scapegoat-like performances (the killing of carnival and similar rituals as a necessary act to ensure fertility and the perpetuation of the agrarian year); and a draft of a social theory of ritualized disorder, which was to be theorized, later and more or less in accordance with Frazer’s formulation, by other scholars working on European folklore, and which is today referred to by the expression ‘safety-valve’ theory (Burke 1978, 185–91; Stallybrass and White 1986; Testa 2014a, 202–24). All of these three elements have been hugely influential in folkloristics, ethnology, anthropology, and the history of religions. In particular, the functionalist interpretation of carnival festivities as rituals of or for fertility (which should properly be called the ‘Mannhardt–Frazer’ hypothesis) would spread widely through Frazer’s popular work, which had a huge influence, although not always in an uncontested manner. Detractors of his theories and methods existed both when his main works were being published and immediately afterwards. Well known, for instance, is Ludwig Wittgenstein’s critique, which did not address the question of festivities and survivals directly, but questioned Frazer’s method as a whole (Wittgenstein 1967).

In Italy, one of the European countries renowned for its long tradition of folkloric studies, Frazer’s theories about carnivals were studied and used early by scholars, but they were made popular mainly by Paolo Toschi, through his very influential book *Le origini del teatro italiano* (The origins of Italian theatre, 1955). Toschi’s interpretation of carnivals and carnival-like festivities and their ritual features borrows heavily from Frazer’s analysis and conclusions (on the extent of this borrowing, see Fresta and Clemente 1984). Conversely, a voice significantly and authoritatively discordant on the relationship between carnival and fertility is that of Arnold van Gennep, a French ethnologist and folklorist who is also considered one of the fathers of both modern folklore and anthropology (see Charuty et al. 2010) and whose theories are still influential, especially in the field of ritual studies. Van Gennep was profoundly sceptical of Frazer’s methods and conclusions, and often criticized his British colleague in his monumental encyclopedia of French folklore—particularly in the book devoted to the ‘cyclical seasonal ceremonies’ (Gennep 1947). He refused a ‘survivalist’ approach on the basis of the assumption that social and cultural facts can exist and live only insofar as they bear an actual function for society; if it were not so, they would not persist as cultural relics, but would be disused and abandoned. Van Gennep’s theoretical framework is differently and more maturely functionalist than Frazer’s. The French scholar also emphasized the scapegoat role of carnival representations and their being a means to a ‘controlled disorder’ for society (the ‘safety-valve’ theory here as well). Also, the agrarian and cyclic dimensions are as present and important in van Gennep as they were in Frazer, but in the speculation of the former there is no hypothesis about the death and resurrection of corn-gods, or the appeal to sympathetic magic, or a catch-all interpretation based on the notion of fertility. Instead, in van Gennep we find a generic assumption of positive and prosocial function and insistence on the importance of the social and psychological aspects of carnival festivals (on these issues, see Testa 2013, 86–94 and 117–24; 2014a, 323–50).

Despite these reservations and critiques, the diffusion of Frazer’s main theses was pervasive to the extent of becoming an interpretative standard—or even a canonical exegesis—for specialists, and even more so for amateurs and cultured but non-academic people, as discussed in greater detail in the following sections.^6^ Nonetheless, its applicability and validity have been addressed numerous times ever since its formulation, and it is today widely regarded as inaccurate or at least not as universally applicable as its progenitor thought.

## Carnival and Masopust in Castelnuovo al Volturno and Hlinsko v Čechách

The ethnographic account and its interpretations that follow are based on my intensive investigations of two revitalized European festivals that have gone through significant transformations over the last few decades.^7^ These investigations took place in Italy (2010–11) and in the Czech Republic (2013–14).

**Figure 1.**
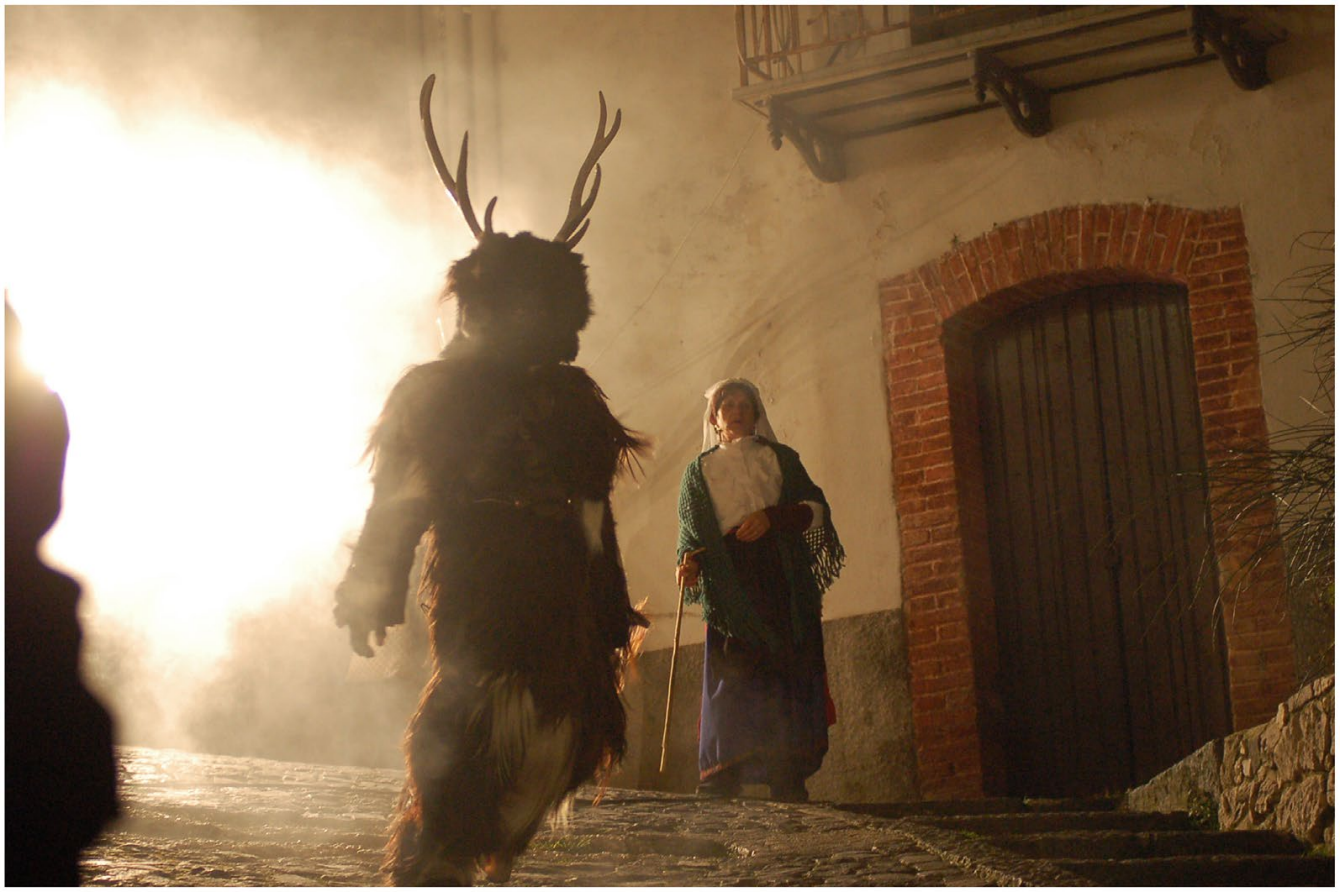
The ‘deer-man’ enters the piazza. Photograph by Aldorindo Tartaglione (2009). Used with permission.

The first festival is the carnival of the man-deer (*Gl’ Cierv* in the local dialect) in Castelnuovo al Volturno, a small village of the Central Apennines. This annual ceremony is characterized by some very archaic features, amongst which is a ritualized pantomime dramatizing the hunt for a man disguised as a deer (see Figure 1). The performance ends with a ‘magical’ ritual act that is supposed to bring fertility to the community. The second case is *Masopust* in the town of Hlinsko v Čechách and surrounding villages, in Bohemia, which is characterized by what in the English-speaking world is known as ‘mumming’: door-to-door processions of masked men who perform dances and other pseudo-ritual actions to ensure, as they claim, good luck and fertility (Blahůšek and Vojancová 2011).

The history of Castelnuovo’s carnival has been thoroughly reconstructed elsewhere (Testa 2014a). The first written source about it dates back to the 1960s, although oral sources make it possible to attest its existence since at the least the second half of the nineteenth century. Like many others in Europe, this festival went through a period of relative neglect and abandonment during the 1950s and 1960s, only to be reborn afterwards, from the late 1970s through the 1990s, when it was revitalized, refunctionalized, and charged with new forms, meanings, and functions.

 The Masopust in Hlinsko is not particularly original in itself, and shows many common features with other carnival-like festivals in the Czech Republic, Slovakia, and elsewhere in Central and Eastern Europe. It is better documented than the Italian case, and, unlike many similar popular manifestations, it was not prohibited during socialist times, although in the Czech Republic, as elsewhere in the socialist world, the official position of the Communist Party towards this kind of tradition was one of discouragement. In any case, this festival also went through a phase of partial neglect during the 1980s, but during the 1990s it acquired a new relevance and popularity, which have since continued to grow. As an ideal end to this process, Masopust in Hlinsko was included on the UNESCO Representative List of the Intangible Cultural Heritage of Humanity in 2010. Regarding the carnival of Castelnuovo, which has also experienced a huge revival over the last years, there are rumours that it too will soon become a candidate for the same recognition.

Among the many changes these two festivals have undergone in recent times, a rather striking one is the emergence—or re-emergence—of beliefs in their power to induce fertility, magically. As a consequence, a set of magical or pseudo-magical acts were invented, re-invented, or re-enacted from times past as a means to substantiate such beliefs. This was a rather uneven process, which happened during a period of great social transformations that occurred in both places between the late 1960s and the early 2000s. A detailed description of the changes in the festivals, the social transformations that determined them, and beliefs and magical acts can be found in the first part of this study, ʻ“Fertility” and the Carnival 1’ (Testa 2017).

What the present article will attempt is to interpret differently the ethnographic data presented in ʻ“Fertility” and the Carnival 1’, but also to present and analyse further ethnographic material from my fieldwork and the scholarly literature on similar cases. More particularly, from this point I will focus on the symbolic sources of the beliefs and practices connected with the festivals and their rituals in Castelnuovo and Hlinsko, and especially on how they have been developed, legitimated, propagated, and maintained during the last few decades and up until today. An account is given of how the ritual of fertility was invented in Castelnuovo, how the fertility-bringing function became the core of the belief described previously, and how it developed to become the ‘official’ interpretation of the festival for different actors, not only locals. I will then present the case of Hlinsko v Čechách.

## Inventing, Reinventing, and Disciplining Tradition

I have already articulated a short genealogy of the notion of ritual fertility in anthropology and the history of religions, insisting on its Frazerian origins and on the persistence, at different cultural levels, of related hypotheses. This persistence of Frazerian themes has been the object of investigation of other scholars as well, who have argued about and demonstrated the success and the longevity of the survival/carnival/fertility complex developed and spread by Frazer. Since then, this ‘complex’ has appeared in many pieces of scholarly, literary, and popular culture,^8^ with or, more often, without the carnival—but this is not significant insofar as carnival for Frazer was only one among many rituals of fertility which had ‘survived’, although in different cultural forms, from ancient times. There was also a time when, in the field of religious studies, the explanation of a ritual on the grounds of its alleged function for bringing fertility was so popular that nearly every performance or rite whose function or provenance was not completely clear was to be associated with fertility. This connotation is in most cases either so obvious that it is banal, or so general that it is vague, or so arbitrary that it is indemonstrable. But let us now return to our case studies and see how these considerations apply to them, starting with the carnival pantomime called ‘Gl’ Cierv’ in Castelnuovo al Volturno.

As demonstrated elsewhere (Testa 2014a, 243–321), even the old rite of Castelnuovo, embedded in the context of agricultural activities, had no fertility function (Testa 2014a, 225–42). The question therefore is: how is it possible that a connotation of fertility, which never existed before, was attached to the revitalized pantomime in the late 1980s, precisely when the activities that should have been associated with this ritual function were dismissed almost completely in the entire area?

Was this trait spontaneously invented by the locals, or did it emerge as a response to changing social and cultural circumstances? The latter explanation can be discarded quickly on the grounds of two main arguments: first, it would have made little sense to enact a rite characterized by very archaic features and connected to a rather elementary religious function for the sake of material activities which were, on the contrary, in the process of being dismissed; and second, even supposing for the sake of argument that a function of fertility induction might have existed (which it did not) before the rite became obsolescent in the 1960s, why should the people of Castelnuovo have abandoned a practice that was so socially significant? Consequently, we cannot but conclude that, even if this function had existed before, it lost its importance quickly; otherwise the locals would have continued enacting the rite that was supposed to sustain it.

Regarding the first explanation mentioned—that is, was this ritual fertility spontaneously invented by the locals?—the answer is again negative. In fact, a very important written source of the period (from 1985 more precisely; see Testa 2014a, 225–42) does not mention fertility or similar notions at all, and also makes it evident that until the mid-1980s there were doubts and conflicts of interpretation about the general raison d’être of the ritual before its abandonment. So, why was it precisely fertility which was chosen as the main emic interpretation of the ritual’s function, soon to become its ‘official’ interpretation? How and why was that gesture of tossing a handful of grain invented, and how is it possible that it soon became the climax of the performance and one of the most important elements of the entire festivity, to the extent that an entirely new belief developed around it?

As already said, the late 1970s and especially the 1980s were the years during which carnival in Castelnuovo regained a new centrality in the social life of the village. This process was partly spontaneously undertaken by some of the more active members of the local community, partly promoted by a non-professional ethnographer active in the region, Mauro Gioielli. Such a process is utterly consistent with the coeval European wave of a renewed interest for rural traditions and folklore. In the early 1990s, then, some important changes happened that were to foster even further the re-enactment of the carnival rite of Castelnuovo: the intervention of a television journalist and amateur ethnographer, Pierluigi Giorgio, and the foundation, in the year 1993, of a village-based Cultural Association (Associazione Culturale ‘Il Cervo’ henceforth ‘AC’) with the purpose of obtaining funding from public institutions to undertake actions such as the safeguarding, promotion, and organization of the carnival.^9^ At the same time, a body of literature started flourishing around and about Castelnuovo’s festival. The AC started to issue a periodical, *Il Richiamo*, to publish news, notices, and interpretations concerning the pantomime. We will come back later to this rich corpus of writing.

Pierluigi Giorgio was hired by the AC to produce a documentary about the rite of Castelnuovo (from the early 1990s, the carnivalesque pantomime is described almost exclusively with the word *rito* in Castelnuovo). He accepts, develops a genuine curiosity for the event, which matches his amateur enthusiasm for ethnology, and starts to work on some changes to make it, in his words, ‘more authentic’. The ‘première’ of the new ‘rite’ under his direction takes place in February 1993. The documentary is published in the same year. What the Castelnovesi witness then is a wholly new thing from what they were used to. A few months later, Giorgio also publishes in the AC’s periodical a detailed account of all the changes he imagined and implemented. The following is a selection of significant excerpts from his text:^10^

I have cleaned up the tradition from the carnivalesque pollutants [*sic*], trying not to alter but to amplify its magical-pagan and religious aspects. [A description follows of many and rather radical changes in the pantomime’s structure, masks, and actions.]

An emphasis has been put on the killing and the revivification of the beast as the peak of the propitiatory rite of fertility.

A final gesture has been added: the deer-man tosses a handful of grains to the winds and to the community to highlight the positive achievement of the rite and the realization of a desire for fertility [*sic*].

All of these elements were immediately and peacefully accepted by the locals, with little or no resistance. Since then, these changes have never been altered; nor has anybody ever proposed to restore the pantomime as it was before Giorgio’s intervention. On the contrary, the gesture that since then has sealed the event is actually, as we have already seen, at the centre of a rather strong religious or pseudo-religious belief. The only one who denounced the modifications of the ritual features of the performance was Mauro Gioielli. In fact, shortly afterwards he wrote an article to expose Giorgio’s blatant alterations (Gioielli 1993). In so doing, Gioielli did not refrain from chastising the villagers who had so light-heartedly accepted such radical changes, which, in his opinion, had profoundly altered both the form and the meaning of the former event. A few years later, Gioielli also published the first book about ‘Gl’ Cierv’ (Gioielli 1997), where he reiterated his criticism of Giorgio’s ‘direction’, and of the new configuration that the ‘rite’ had consequently acquired. As a reaction, Giorgio sued him, but also withdrew from any personal or professional involvement with Castelnuovo and its carnival.

The struggle between Giorgio and Gioielli was also a political one: it was fought over which interpretation of the rite was to be considered ‘right’, hence legitimate, and therefore to determine who was capable of being the voice of the ‘authentic’ tradition. At the same time, they were trying to impose their interpretations on the locals, albeit implicitly, and to ‘purify’ the tradition—‘clean it up’, in Giorgio’s words—from the pollution of the modern times, thus restoring its pristine form and meanings. Their view of tradition is that of something mutable and even too prone to change, something that should therefore be disciplined to express its authentic features appropriately. The AC supported this process, because it legitimized these claims through publications (i.e. it made their interpretations more valid and reliable because of their being written and published), but especially through a process of ‘crystallization’ brought about by bureaucratization and institutionalization. In fact, after 1993 the AC became the only agent capable of funding, organizing, and actually making the event happen, hence controlling every single aspect connected with it. Thus the once spontaneous carnival of Castelnuovo and its many features were ultimately disciplined.

But let us return to Giorgio’s interpolations and supplements to the ‘rite’. Even through a superficial reading of the lines cited earlier, their Frazerian air is clear. Reading the works of his opponent, Gioielli, however, it is likewise plain that his main source of inspiration was also Sir James Frazer, albeit better read and assimilated. In both Giorgio and Gioielli we find the insistence on ritually-fostered fertility, although differently expressed. In both we find the importance of the killing of the sacred animal, the rite marking a passage to a new season, the opinion about this particular carnival being the survival of an old pagan festival (and this in spite of the village being founded during the Middle Ages and the first written source about its carnival no earlier than the 1960s).

These same opinions and interpretations appear in all publications of the AC: its periodical, books, brochures, and videos, which since 1993 have circulated in the entire region and have been avidly read by the villagers and whomever is interested in their carnival. It should also be noted that the great majority of these pieces of writing have been published by people with little or no training in history, anthropology, and folklore. In this corpus of writings the most common adjectives that qualify the pantomime are the following: totemic, pagan, shamanic, Dionysian, archetypal, mysterious, primordial, prehistoric, very ancient (*antichissimo*), millennial. None of them captures one single trait of the pantomime, and yet they have become canonic, and shape the very opinion of both the locals and outsiders.

As in many other European contexts, the local examples of popular culture are permeated with romantic properties, and believed, rightly or not, to bear genuine but also sometimes mysterious meanings, and to be ancestral, according to an equation between antiquity and authenticity that will be discussed later.^11^

All of these ideas, which were surely not common among the villagers before the 1980s, were slowly assimilated through a sort of implicit acculturation. This was made possible by the fact that the Frazerian changes in the event and its Frazerian interpretations actually intercepted imaginaries, narratives, and poetics of history that were at work throughout Europe in precisely those years, as an abundance of works has demonstrated. The fact of their being introduced and promoted by individuals provided with a higher social and cultural capital (Bourdieu and Richardson 1986) than most of the villagers allows us to consider them etic, at least to a certain extent.^12^ However, they quickly turned emic, and started to be shared by the entire community and to circulate especially by means of the work of the AC and its periodical. This happened quite smoothly and quickly, partly because of the greater power of persuasion (grounded on a higher cultural capital) of Giorgio, Gioielli, and some of the authors who published in the periodical, and partly because one may assume that a certain imaginary complementary to the Frazerian one did already exist among the villagers when they started to be influenced by Gioielli.

In Hlinsko v Čechách and in the surrounding villages where Masopust is performed, the local carnival has acquired, in the last two decades, just as in Castelnuovo, a renewed importance, up to the point that it is widely regarded—and mostly participated in—by the locals as the most significant social event of the year. Unlike in Castelnuovo’s carnival, in Hlinsko the fertility-inducing function of the Masopust rituals is a longstanding and well-documented feature, as already argued in ‘“Fertility” and the Carnival 1’ (Testa 2017). What, then, is ‘Frazerian’ in Hlinsko’s tradition?

First, the insistence on magical fertility in the time of clinical medicine, and after decades of socialist rationalism, industrialization, and de-ruralization, is itself a rather ‘Frazerian’ feature. In other words, the spontaneous belief recorded by folklorists as early as the first half of the nineteenth century, and the strong social function it bore for the locals living mostly in pre-modern conditions, cannot obviously be coupled with the contemporary one. So who insists on fertility (*plodnost* in Czech) today, and why?

On the one hand, there is obviously a cultural continuity that should not be underestimated, because, in a way, fertility-bringing rituality seems to have never really abandoned these lands. On the other hand, and perhaps most importantly, Masopust fertility has been the object, just as in Castelnuovo, of an etic interpretation and ‘rehabilitation’. It has also been institutionalized (we are about to see in what terms), together with the entire tradition to which it is bound.

Fertility is present in the ‘official interpretation’ of the local Masopust developed in the book written by the director of the Masopust museum and chief of the local bureau for the preservation of national cultural heritage, Ilona Vojancová (Blahůšek and Vojancová 2011, 78–79). Ilona Vojancová is also a very active person in the local community. Besides profusely publishing works of popularization about Masopust in the area of Hlinsko, she is involved in many social and cultural activities, and is also a tireless promoter of the local heritage. Her very high social and cultural capital, which translates into the almost unanimous high consideration people of Hlinsko have for her, has also determined her ‘political status’ as the holder of the correct interpretation of the local tradition, up to the point that many informants, when asked about the Masopust, simply answer: ‘ask Ilona, she knows’. As a professional ethnologist, a bureaucrat, and the main interlocutor of UNESCO, she was also in charge of producing the official application documentation for the prestigious recognition. In this document, we read that ‘the actions of the masks are associated with magically securing fertility’.^13^

Hence, anachronistic as it may seem, the evocation of magical fertility is nevertheless present and sound. It is, however, possible to find more genuine Frazerian themes in the UNESCO documents, the cultural products about Masopust (brochures, books, guides for tourists, videos), and, especially, in the local imaginary.

According to the many records gathered among my informants, Masopust is often referred to as a very ancient or, alternatively, pagan festival which has survived both the prohibitions of the Church and of Communism. One of its main purposes would be to mark the change of the season: rid the community of the winter, renew the earth, and bring good fortune by means of agrarian magic connected to the seasonal cycle. Its Bacchic or Dionysian nature is mentioned often, at times in relation to the ritual killing of the masked mare, considered the crucial moment of the entire event.

Today, these Frazerian themes are mainly present in and diffused by the aforementioned products of mass diffusion, which both sustain them and are sustained by them.^14^ In fact, only some of the previously mentioned features appear in folkloric literature prior to the 1990s, whereas today they are widespread among locals, the majority of whom have no knowledge whatsoever of Sir James Frazer, his literature, or the scholarly literature which has made use of his interpretations.

It must be said, however, that in Hlinsko (but to a lesser extent in its surrounding villages), beliefs and opinions about Masopust differ more than in Castelnuovo, with some categories of people (usually individuals nostalgic for the regime or with a higher education) being less inclined to appreciate Masopust and/or the imaginary with which it is usually associated. In the Czech case I have also noticed a more critical spirit amongst the locals, and a tendency to distinguish between the more radical notions of ‘authentic’ tradition as opposed to ‘made up’ tradition—the emic categories being *tradice*, ‘tradition’, and inauthentic things, *na oko*, ‘for the eye’ (i.e. made up just to entertain). Nevertheless, the Frazerian themes are widespread, especially among those who participate in the Masopust performances.

In Castelnuovo, the synergy between the AC and its publications, the journalist, the local ethnographer, and the imagination of the villagers shaped a new tradition, fortified a collective imaginary, and created not only a new interpretation of the rite and its function, but also a brand new pseudo-religious belief in its magic and effectiveness. Likewise, in Hlinsko this synergy took place between several institutions and publications, the work of an ethnologist and bureaucrat, and the locals’ beliefs. If in Hlinsko the tradition was less manipulated formally and semantically by outsiders—and if the belief in magical fertility was less invented than re-enacted—it nevertheless acquired new Frazerian properties as a result of the renewed interest in it after the fall of the socialist regime. Furthermore, after Communism the local tradition in Hlinsko was institutionalized and disciplined exactly as it was Castelnuovo, but with the contribution of a much more important and powerful institution than a village-based cultural association: UNESCO.

The coming of UNESCO to Hlinsko did not happen without tensions, especially when the nomination was proposed and then immediately after it was accepted. Many of the most active members of the local community organizing and participating in the Masopust performances expressed concerns about the possible changes that UNESCO could bring to their tradition and about the interventions of externals in their internal affairs. Some started to question themselves, one another, and Ilona Vojancová about the UNESCO candidature and nomination, thus expressing a manifest worry about their ‘cultural intimacy’ being exposed.^15^ From this resulted a certain ambivalence in the townsfolk—or at least among those more involved in the tradition-making—between sentiments of pride for the international recognition and fear of ‘cultural pollution’ or external interferences. When, however, it appeared clear that UNESCO would have no real impact on the actual organization and implementation of Masopust, and that actually the UNESCO experts demanded people keep on doing as they had always done, even the purist traditionalists accepted more or less light-heartedly the presence of such a cumbersome new host. Today, the power confrontations and political positioning within and around the Masopust in Hlinsko and the UNESCO label are more articulated. These aspects will be fully developed in another, forthcoming article,^16^ because they deserve a full-length description as well as a sophisticated interpretation concerning cultural-heritage-making and the contextual general politics of culture that cannot be developed here.

It is in fact now necessary to reflect further on how the production, circulation, and negotiation of meanings that we have seen at work so far can be thought of in more theoretical terms.

## Popular Frazerism: Cultural Bricolage and Circulation

Once, in Castelnuovo, an informant told me: ‘It is only thanks to the scholars (*studiosi*) if we people from Castelnuovo (*Castelnovesi*) have come to understand the importance of our rite (*rito*)’ (original quote in Testa 2014a, 457). It is true, in fact, that people like Pierluigi Giorgio and Mauro Gioielli, and others, have contributed substantially to the construction of the meaning and the imaginary of the carnival in Castelnuovo as observable at present. Very similar statements I have heard many times in Hlinsko, especially with regard to the work of Ilona Vojancová.

At the same time, the interpretations and opinions of those with a higher cultural capital than the tradition-holders had to meet the expectations of the latter, who in their turn had to possess a set of collective representations (here called an ‘imaginary’) capable of understanding, assimilating, adapting, and expressing such new interpretations and opinions. When, besides, these mechanisms involve not only individuals, no matter how influential they are, but also institutions, with all the symbolic and political power they bear in Europe, then it is clear to what extent these representations and narratives can become socially significant.^17^

There are numerous anthropological and historical accounts of phenomena of cultural circulation and reinvention or manipulation of folkloric traditions in Europe—whether they deal with the popular reappropriation of elements of ‘high’ or official culture, or vice versa, or cases of continuous recycling of knowledge.^18^

In this respect, the cases described by Andrew Lass, Matt Hodges, and Michael Herzfeld are particularly significant because they are quite convergent with mine (Lass 1989; Hodges 2011; Herzfeld 1982). Even more consistent is the case of ‘Tarantism’ in southern Italy described by Giovanni Pizza in a piece that highlights and interprets the dynamics of social circulation, reinvention, and reappropriation of what was an anthropological theory (developed in the 1950s and 1960s by the Italian anthropologist Ernesto de Martino in a series of groundbreaking works; for example, Martino 1961). It was precisely this theory that, through those processes of cultural reworking and bricolage, transcended the boundaries of the scholarly ivory tower and percolated into a network of mass post-modern representations. Through this process, the said theory was transformed eventually into a set of widespread social narratives used and consumed for political, economic, and identity purposes (Pizza 2004).^19^

The expression ‘cultural bricolage’ is used to signify the construction of imaginaries, taxonomies, narratives, or more or less coherent sets of representations on the basis of elements and features taken from culturally and socially available symbolic sources. This short definition elaborates further that of Fiona Bowie, who writes that ‘The term *bricolage* has been widely adopted within anthropology to refer to the creation of symbolic structures from a variety of culturally available symbols’ (Bowie 2006, 70). My use of this notion depends mostly on Claude Lévi-Strauss’s theorization of native classificatory systems, but also, and perhaps especially, on the observations about its theoretical and methodological utility by Peter Burke (Lévi-Strauss 1962; Burke 2008, 100–101).^20^ The notion of cultural bricolage is akin to that of acculturation, but it is more convincing than the latter because it avoids the idea of a passive and immediate passage of elements from a social group to another, pointing out, conversely, the adaptability and creative agency inherent in the composition and invention of cultural features from a variety of symbolic sources, social dynamics, and historical processes.

In the cases studied by Lass, Hodges, Herzfeld, Pizza, and myself, among others, what we see at work is a continuous loop of knowledge that involves different types of social agents: individuals, groups, and institutions. Local sentiments and practices merge with translocal imaginaries and academic notions through dynamics of circulation and reappropriation that become components of an engine which, in the end, produces social poetics and practices that fuel *agency* at a community level, and that contribute to the self-representation of the community itself, as I have tried to demonstrate elsewhere (this is one of the main conclusions of Testa 2014a) and will briefly argue in the last section of this study.

The synergistic interactions of these different levels of meaning and social practice can be conceptualized as a form of collective cultural bricolage, largely unconscious but nonetheless provided with an effective power to change the traditional practices, give them new meanings, and shape the social representations and narratives on which they are based. These dynamics have happened under the sign of a resurgence of the need for historicity and poetics of the past gravitating around traditional practices—as well as around the very notion of tradition. With regards to this, little or no difference exists between the two contexts at the centre of my ethnography. What I have in fact recorded in both Italy and the Czech Republic is a peculiar way in which correlated notions of antiquity, authenticity, and ‘traditionality’ are thought, mobilized, used, and experienced in everyday life by the local people, mainly but not exclusively on the basis of their respective carnival events. These notions have been at work and have functioned synergistically since the time of the revitalization of the rituals, which is to say, for several decades now, acquiring even more importance as the material conditions of life of both communities worsened.

But why do I propose to name this complex set of connected social phenomena and dynamics ‘popular Frazerism?’^21^ The reason is that in the kind of cultural circulation and bricolage at work in my cases, as well as in others that can be compared thanks to the existing literature, the main symbolic source of inspiration for the emic understanding of rituals and festivals (as well as for the local aesthetic flair connected with them) has been, as this work has tried to demonstrate, Frazer’s theses on European agrarian festivities and folk rituals, or rather a popularized version of said theses. For instance, this includes those theses concerning the notion of ritually-fostered fertility (or *propiziazione*, as it is called in Castelnuovo), agrarian magic, the supposed pagan origins of carnival and Masopust—their being a survival of ancient rituals (at times considered of presumed unfathomable antiquity or even prehistoric).^22^ In this respect, the case of Castelnuovo is particularly striking because of at least two other factors: first, the Frazerian ‘agrarian magic’ at the core of the pantomime ‘Gl’ Cierv’ results not, as we already know, from a spontaneous popular inventiveness, but from an operation of cultural bricolage promoted by a single social actor—moreover, one external to the local community, and accepted by the vast majority of the locals. This dynamic eventually led to the shaping of a brand new ritual magical act and also to the formation of the belief in its effectiveness, which is furthermore a rather striking example of the emergence of a new symbol through an act of ritualization promoted by a social agent external to the locals and to the ritual performers.^23^ Second, it can be demonstrated easily through documentation that virtually all of the interpretations proposed and diffused by Pierluigi Giorgio and Mauro Gioielli (in spite of their rivalry), as well as in the numerous publications of the AC, are based on Frazer’s *The Golden Bough* (1922). However, the critical knowledge, understanding, and usage of Frazer’s actual theses by these different social actors can vary significantly (Gioielli’s Frazerism is sound; conversely, the great majority of the articles published in the journal *Il Richiamo* are signed by authors with little or no education in history, folklore, or anthropology).^24^

## Reconfiguring Tradition

Popular Frazerism is one of the possible modalities to ‘normalize’ tradition, encapsulate its interpretations, and discipline the potential conflicts of interpretations by bringing them back to a well-established and vastly spread imaginary of antiquity, magic, and primitiveness; an imaginary which fosters ritualization and creates sentiments of authenticity, cultural purity, and community identity by means of a veritable social mythopoesis.

The informant’s sentence quoted at the beginning of the previous section represents a widespread opinion among the locals in both Castelnuovo and Hlinsko. Besides its being representative of the mechanisms at work in the process of cultural circulation between social classes, this statement highlights another dimension of the reconfiguration of tradition as it has been conceptualized so far: the ‘regime of truth’ (Foucault 1977; Weir 2008) that arises from a social narrative like the discourse on tradition. Since the 1980s in Castelnuovo, and since the 1990s in Hlinsko, the appearance in the field of the local tradition of experts, scholars, functionaries, journalists, and other categories of people, with social/cultural capitals generally higher than those of the people actively involved in the festivities, has determined a demarcation between those legitimized to propose their discourse about the performances and the festivals, and therefore to say something ‘true(r)’ about them, and those who have somehow become more ‘passive’ recipients and reproducers of said discourses.^25^ In Castelnuovo, this has occurred not completely peacefully, as is demonstrated by the quarrel between Mauro Gioielli and Pierluigi Giorgio, who tried to discredit one another’s interpretations and in so doing ‘hegemonize’ the discourse about the pantomime. At stake was the symbolic capital of the festival, a capital to be used, among other aims, for social recognition, personal interests, and even political power, as argued elsewhere on the grounds of empirical evidence from the fieldwork (Testa 2014a, 521–36; 2014c).

Hence, the tradition becomes an arena (or a *champ*, according to Bourdieu 1994) where different social claims, personal interests, and community needs are expressed and negotiated. In this arena, a special role is played by scholars and students, whether professional or not (and sometimes plainly self-proclaimed so). This is especially significant in the cases of ‘folkloric’, historical, or other sorts of public rituals characterized by a strong diachronic dimension; those cases, in other words, where local and amateur historians and ethnographers can act as mediators between academic and popular interpretations, and embody in themselves this mingling. It is they who foster and promote cultural circulation, and it is they who, in the process, become the authorities—and sometimes the masters—of local history and tradition, not seldom with the help of cultural institutions and local influential people like entrepreneurs or politicians (Hodges 2011; Macdonald 2013; Palumbo 2006, 233–321; Testa 2014a; Sagnes 2002).^26^ Thus, natives, visitors and tourists, experts and scholars, bureaucrats, and all other possible categories of people who participate in or gravitate around the local tradition—which may be, or may not be, or may not *yet* be officially recognized as ‘cultural heritage’—get tripped up in the invisible tangles of power and of discourses and regimes of truth.

In small, marginal communities in search of identity and visibility, local traditions, especially in their festive or public versions, offer a strong cultural glue, sometimes a veritable raison d’être for the local society, often in response to disruptive social changes brought about by late-modern processes (see Testa 2017). The power of tradition makes its set of interpretations and representations so significant as to become social narratives, often also charged with religious connotations.

This conviction brought me to re-evaluate the power of local tradition and to orient my research, in the last few years, towards the understanding of the social construction, circulation, perception, and consumption of things deemed traditional in rural or semi-urban European contexts.^27^ These things all share at least one component: the fact of being linked to an actual or imagined past. The relationship to a past, or rather continuity along the axis of time—whether emically conceptualized as history, heritage, tradition, or differently—is the common denominator of the otherwise vast and diverse set of social facts, phenomena, and processes that qualify as traditional. In the case of folkloric events like carnivals, the best type of past, in a manner of speaking, is the antique time of pagan festivals, according to the ‘popular Frazerism’ explored here. It is this ‘sense of the antique’, more than any possible and actual antique feature—which can be easily altered or even invented—that permits binding a tradition and the people who practice it to a past which can be used to enhance sentiments of belonging, community, and identity. This poetics of time produces, in its turn, symbolic depth, which fuels social memory and usually translates into sentiments of typicality and authenticity.^28^ As Saša Poljak Istenič has put it, ‘in this sense “traditional” means not only old, but also original and authentic’ (Istenič 2012, 79).^29^ The problem is that ‘authenticity’, unlike historicity, is never a quantifiable or verifiable given, but precisely a poetics, as was already claimed thirty years ago: ‘what is historical and typical is authentic, and it is assumed that authenticity is objectively ascertainable’ (Handler 1988, 200).^30^ Thus one can explain the emic usage of adjectives like ‘very ancient’, ‘antique’, ‘pagan’, or even ‘prehistoric’: the equation at work is that the more remote the evoked past is, the more ‘authentic’.

Through this eminently socio-cultural mechanism, sentiments and representations of typicality, authenticity, historicity, heritage, and tradition start to function synergistically, shaping a wider framework of social imagination (collective memory, poetics, and imaginaries).^31^ With this last observation, my reflection joins that of Sharon Macdonald, who has recently theorized about similar problems in a wider comparative fashion. She has named this ‘family’ of social phenomena ‘European memory complex’, seen as ‘a shorthand for something like “the memory–heritage–identity complex” for these are all tightly interwoven’ (Macdonald 2013, 5).^32^

In conclusion—and here the conclusions of the sibling article, ʻ“Fertility” and the Carnival 1’, are also evoked—it is interesting to notice once again how popular Frazerism, together with being a paradigmatic type of culturally-bricolaged tradition and a declension of a post-modern romantic imaginary of magic and primitiveness, can also become the very source of religious beliefs,^33^ in a fashion that bears similarities to other cultural and religious phenomena such as Neopaganism.^34^

Popular Frazerism is one of the modalities to reconfigure tradition in Europe today, allowing individuals and communities to imagine and build up their past, manufacture cultural heritages, shape feelings of belonging, and discriminate between authentic and inauthentic objects and practices. Moreover, it can constitute one of the tools for the construction of local identities as well as a viable cultural device to expose those very identities and make them circulate in the media, envisage different modes of social representation, and experience new forms of religiosity.

## Notes

1 The historical, folkloric, and anthropological literature about European carnivals is enormous. I have reviewed and critically used a substantial part of it in my last book (Testa 2014a). Those who do not read Italian will find some useful references in Testa (2014b, 2014c).

2 The notions of ʽemicʼ and ʽeticʼ are used according to Kenneth Pike’s theories, as transposed into anthropological methodology and used for interpretative purposes by Marvin Harris (Pike 1967; Harris 1976). ʽEmicʼ refers to beliefs or belief systems of a given group as differentiated from scholarly interpretations and categorizations of those same beliefs, for which the word ʽeticʼ is used.

3 Reference will be made only to Frazer’s major work, *The Golden Bough*, and especially to its Abridged Edition (Frazer 1922). *The Golden Bough* was originally published in 1890, and a complete twelve-volume edition published between 1906 and 1915. Nevertheless, the shortened edition is ʽby far the most diffused and influentialʼ (Clemente, Dei, and Simonicca 1984, 5).

4 I use the word ‘propitiate’ (Italian *propiziare*, Latin *propitiare*) and its derivative ‘propitiatory’ in a slightly different way from the common use in anthropology and the history of religions, in order to reflect the emic use of my Italian informants. In Italian it is possible to say ‘to propitiate fecundity (or fertility, or the harvest)’ in the sense of making ‘something’—whether thing, or god, or man—propitious or favourable, although the most common connotation is that of appeasing the god(s).

5 The functionalist hypothesis of the propitiatory nature of seasonal rituals practised in rural Europe to promote or invoke the fecundity of the earth did not originate with Frazer. In fact, he developed it on the basis of the work of a German scholar, Wilhelm Mannhardt, who had proposed a similar interpretation in his book *Wald- und Feldkulte* (Cults of forest and field, 1875–77).

6 Italian anthropologist and historian of Frazer’s thought, Fabio Dei, has written that ʽFrazerism was evident especially in the tendency to interpret whichever folkloric element either by evoking the laws of sympathetic magic or by considering it a survival of ancient agrarian rites. Folklore as a discipline has been deeply influenced by this kind of approach … Studies on superstition, festivals and popular theatre, on Carnival, games, witchcraft, popular medicine and on many ceremonial facts have not been able to avoid the influence of Frazerian thesisʼ (Dei 1998, 411; my translation). Critical analysis of Frazer’s influence in Western contemporary cultures (academic, literary, and popular) has been undertaken in many works. For Italian contributions, see Dei (1998), Dimpflmeier (2014), Clemente, Dei, and Simonicca (1984), and Dei and Simonicca (1998). See also Testa (2012; 2014a, 449–58).

7 An overview of the changes in the forms, functions, and meanings that European festivals and public rituals have undergone over the last few decades, especially in rural and non-urban areas, is given in Testa (2017).

8 Throughout the twentieth century, Frazer has inspired a great variety of genres in popular culture in Europe and in North America. His influence can be found in cinema, in many sub-genres of contemporary music (world music, folk, and metal), and in products of ʽhigh cultureʼ—literature, for instance. On the persistence of Frazer’s influence in the twentieth century, in spite of his fading influence in anthropological science, see Dei (1998) and Dimpflmeier (2014), and the literature cited therein.

9 This article will not explore the many implications related to cultural institutionalization, bureaucratization, patrimonialization, politicization, mediatization, and touristification of the local tradition brought about, mainly but not exclusively, by the Associazione Culturale ʽIl Cervoʼ (see, rather, Testa 2014a, 405–536; 2016a).

10 All these quotes are translated as faithfully as possible from Giorgio (1994).

11 In this respect, Frazerism can be thought of as a variety of romanticism or post-romanticism.

12 This scenario matches the consideration by Andrew Lass: ʽThe ethnographer’s and folklorist’s presence on the scene is but a new chapter in the history of mediation between “high” and “low” culture. This fact must be seen in the context of a wider perspective: the gradual disappearance of a social reality (the preindustrial, rural world) and the heightened interest in its culture are parts of a single political and cultural processʼ (Lass 1989, 10).

13 Quoted from the official UNESCO candidature document, Nomination file n. 00397, 2010, which can be accessed online: http://www.unesco.org/culture/ich/en/RL/shrovetide-door-to-door-processions-and-masks-in-the-villages-of-the-hlinecko-area-00397. Compare Lass’s description of the way disused local traditions (specifically Czech traditions) had been re-enacted during the 1980s. He reports the following record from one of his informants: ʽThis year, following our book [a book written by a local folklorist], on several occasions during the celebrations of Lent, authentic scenes of ancient spinning rituals with songs, pastimes, customs and games were performed at midnightʼ (Lass 1989, 13). In the ethnographic cases under discussion in this article, both Mauro Gioielli and Ilona Vojancová can also be thought of as cultural mediators between the world of the academics and that of the natives; mediators possessing a significant power of persuasion.

14 A good example is a documentary produced and broadcast by the main national broadcaster (Česká televize 2012). It mixes reliable and fictional/popularized information, with the aim of meeting the taste and expectations of a broad public. As can be expected, Frazerian themes can be found in abundance.

15 The expression ʽcultural intimacyʼ is used in the sense made common in anthropological studies by Michael Herzfeld: ʽthe recognition of those aspects of a cultural identity that are considered a source of external embarrassment but that nevertheless provide insiders with their assurance of common socialityʼ (Herzfeld 1997, 3). Similar cases in which the UNESCO nomination or list inclusion gave rise, in certain segments of the local community and for varying motivations, to criticism, resistance, or even refusal are reported in Bendix et al. (2009), Brumann (2013), Fournier and Bortolotto (2011), Kuutma et al. (2007), and in several papers published in Adell et al. (2015).

16 The article is currently in preparation. Its provisional title is ʻMasopust Revisited: UNESCO, Postsocialism and the Politics of Tradition in a Bohemian Townʼ.

17 Here I refer not only to schools, museums, and national or even transnational bureaux and agencies (like UNESCO) dealing with cultural heritage and the politics of culture in general, with their enormous power of implementing top-down social policies (as with Hlinsko), but also to the more community-level institutions, like cultural associations (as with Castelnuovo). The literature on UNESCO and other agencies dealing with culture and heritage is very rich (see, among many others, Adell et al. 2015; Akagawa and Smith 2009; Brumann 2014; Hemme, Tauschek, and Bendix 2013; Palumbo 2006; Testa 2016a). Much less abundant are the works on the role of cultural associations in shaping knowledge, ideas, and poetics of tradition and heritage, especially in small, rural contexts. An exception is Hodges (2011). See also Palumbo (2006), Pizza (2004), and Testa (2014a).

18 This expression is by M. Carneiro da Cunha, quoted in Bortolotto (2011, 24). A selection of particularly significant ethnological and historical accounts includes Burke (1978, 23–64, 91–115 and 250–59), Ginzburg (2009), Goody (2001), Handler (1988), some of the case studies in Hobsbawm and Ranger (1983), and Sahlins (1993).

19 As L.-S Fournier has argued referring to a similar French case, traditional festivals and their ritual features can today acquire new social functions and meanings ʽplus en raison de [leur] force d’évocation dans l’imaginaire global contemporain que pour [leurs] qualités culturelles ou pour [leurs] rôle social et historique localʼ (more because of their evocative force in the global imaginary than because of their cultural qualities or their local social role) (Fournier and Bortolotto 2011, 152). Clemente (2005) also contains illuminating considerations about the same problem.

20 Discussions of cultural bricolage can be found in Macdonald (2013), Handler (1988), Hobsbawm and Ranger (1983), and Sahlins (1993); more specifically, in relation to festive events and popular culture, in Bravo (2005) and Burke (1978, 179–200).

21 I first used this expression in Testa (2014a, 545). I partly owe it to Paola de Sanctis and her work on ʽ*frazerismo diffuso*ʼ (widespread Frazerism) in the 1980s (Sanctis 1984). Her intuitions were clever, but she did not develop them further, probably because they were not founded on any intensive ethnographic investigation. The notion was again rediscovered and rediscussed later (Dei 1998; Clemente, Dei, and Simonicca 2008). These works, however, deal mostly with the presence and the influence of Frazer in ʽhigh cultureʼ (Dei 1998) or with his intellectual and theoretical legacy in anthropology (Dei 1998; Clemente, Dei, and Simonicca 2008).

22 Castelnuovo and Hlinsko are far from being isolated. It is actually possible to find all or some of these traits in many European ethnographic records. Besides the examples already cited, see Ariño and Lombardi Satriani (1997), Creed (2011), Fournier and Bortolotto (2011), Grimaldi (2003), Gunnell (2007), and Sisto and Totaro (2012). Popular Frazerism seems to be a pan-European phenomenon just like the diffusion of carnival festivities and the use of fertility rites in pre-modern times.

23 This process was described in greater detail in the first part of this study. For the critical notion of ʽritualizationʼ, see Bell (2009, 88–93 and 197–238); for a discussion on why and how it can be applied to the cases discussed in this paper, see Testa (2014a, 56–77 and 499–510).

24 It has been written that ʽFrazer’s theses have held a popular grip, so to say, on non-specialist scholarsʼ (Dei and Simonicca 1998, 23). Often in the non-specialist literature about Castelnuovo’s carnival, Frazer is not openly evoked or cited, yet in it the reader can find expressions such as (I quote from the Italian) ʽancient fertility riteʼ, ʽmagical agrarian riteʼ, ʽthe killing of the sacred (or totemic) animalʼ, and the like. The ʽFrazerian airʼ that characterizes them has become a common trait of the popular interpretations of popular culture.

25 This demarcation could be conceptualized in terms of the appearance, on the horizon of the discourses about the carnivals of Castelnuovo and Hlinsko, of a ʽgreat traditionʼ (originally more learned, written, and etic) as opposed to a ʽlittle traditionʼ (more popular, mainly oral, and emic) embodied in those who simply accepted and reproduced the former (Goody 2001).

26 Very pertinent is the article by Hodges and that by S. Sagnes on ʽthe past of the local historiansʼ, where she highlights the narrative strategies through which ʽlocal historiansʼ build up the past and shape the historical imaginary of small, marginal, and rural communities. In Sagnes, the adjective ʽlocalʼ indicates the fact of being from the same locality at the centre of one’s scholarship as well as being a non-professional historian (Hodges 2011; Sagnes 2002).

27 For a critical theoretical discussion on the notion of ʻtraditionʼ, see Testa (2016b).

28 Nathalie Heinich has written that ʽl’ancienneté est une valeur éminente: elle s’ajoute à la valeur d’authenticité en rallongeant le temps dans lequel s’inscrit le lien avec l’origine de l’objetʼ (antiquity is an eminent value: it increases the sense of authenticity by extending the timespan in which is inscribed the connection with an object’s origin [actual or presumed, I would add]) (Heinich 2012, 29).

29 An Italian ethnologist of Sicilian traditions has concluded, in his turn, that ʽThe discourse about the “ancient” origins is invariably present in the promotion of rural Carnivals … Every Carnival is therefore supposed to be ancient, unique, and originalʼ (Buttitta 2010, 223; my translation). Another Italian anthropologist has more radically concluded that ʽin marginal contexts the antiquity of the festival is a very important element. Why does an “ancient” festival function more efficiently in the construction and the maintenance of the locality? Because the stretching of the temporal depth of the festival means the widening of its symbolic density and stratificationʼ (Faeta 2005, 163).

30 On the notion of—and the search for—authenticity in folklore more generally, see Bendix (1997). The relationship between memory, history (historicity), and social construction is a wider topic that cannot be developed any further here (for the recent debate in anthropology, see Nic Craith and Fenske 2013; Macdonald 2013, 5–21 and 109–36).

31 It goes without saying that with these considerations I am not denying the diachronic dimension of certain social facts (their *real* historicity). On the contrary, in another article I have strongly advocated a methodological ʽmedianʼ or third-path way in the study of tradition that places itself halfway between an utterly deconstructive attitude (which considers tradition as a spurious and made-up social fact and an ʽinvention of modernityʼ; Palumbo 2006, 21) and a less critical one (which gives more credit to the genuineness and veracity of traditional objects and is less concerned about their deconstruction)—see Testa (2016b). The first attitude is usually representative of post-modern anthropology, whereas the second can be more easily found in folkloristics.

32 Cultural heritage is one of the possible ways of institutionalizing this complex in order to meet ʽofficiallyʼ what Christoph Brumann has recently called the ʽpopular demand for historical veracity and authenticityʼ (Brumann 2014, 180–81). The problem of (intangible) heritage as a form of cultural institutionalization and ossification of tradition has been insisted upon by almost all anthropologists and ethnologists working on cultural heritage, so this aspect need not be emphasized further here.

33 In a way, popular Frazerism as I conceive it is also characterized by religious or pseudo-religious traits, and could therefore also be regarded as a possible tool for the shaping of a vernacular religion (Primiano 1995). In Hlinsko, this would be a form of folk religious belief in an otherwise highly secular context; in Castelnuovo, a local variation of Catholicism impregnated with magical elements, which are at times overtly conceptualized by the locals as ʽpaganʼ without this posing any particular doctrinal problem for them.

34 An example of cultural circulation and bricolage very similar to those described in this article, also involving the (re)invention of tradition, especially festive traditions, but on the other hand leading to genuinely religious (Neopagan) beliefs and practices, is described and analysed in a masterly manner in Hutton (2008). Here this parallelism is not postulated *ex silentio*: both popular Frazerism and Neopaganism have in common the longing for authenticity; they both emphasize the importance of rituality and seek—or try to reproduce—their original and pristine form; stress the connection with an ancient past; postulate a strong link between community and traditions; and believe in the ʽpollutingʼ charge of modernization and modern processes in spite of the very fact of their being themselves modern phenomena.
